# OUHP: an optimized universal hairpin primer system for cost-effective and high-throughput RT-qPCR-based quantification of microRNA (miRNA) expression

**DOI:** 10.1093/nar/gkab1153

**Published:** 2021-11-25

**Authors:** Fang He, Na Ni, Hao Wang, Zongyue Zeng, Piao Zhao, Deyao Shi, Yinglin Xia, Connie Chen, Daniel A Hu, Kevin H Qin, William Wagstaff, David Qin, Bryce Hendren-Santiago, Sherwin H Ho, Rex C Haydon, Hue H Luu, Russell R Reid, Le Shen, Hua Gan, Jiaming Fan, Tong-Chuan He

**Affiliations:** Departments of Nephrology, Gastroenterology, Laboratory Diagnostic Medicine, and Orthopaedic Surgery, the First Affiliated Hospital of Chongqing Medical University, Chongqing 400016, China; Molecular Oncology Laboratory, Department of Orthopaedic Surgery and Rehabilitation Medicine, The University of Chicago Medical Center, Chicago, IL 60637, USA; Ministry of Education Key Laboratory of Diagnostic Medicine, and the School of Laboratory Medicine, Chongqing Medical University, Chongqing 400016, China; Molecular Oncology Laboratory, Department of Orthopaedic Surgery and Rehabilitation Medicine, The University of Chicago Medical Center, Chicago, IL 60637, USA; Ministry of Education Key Laboratory of Diagnostic Medicine, and the School of Laboratory Medicine, Chongqing Medical University, Chongqing 400016, China; Molecular Oncology Laboratory, Department of Orthopaedic Surgery and Rehabilitation Medicine, The University of Chicago Medical Center, Chicago, IL 60637, USA; Ministry of Education Key Laboratory of Diagnostic Medicine, and the School of Laboratory Medicine, Chongqing Medical University, Chongqing 400016, China; Departments of Nephrology, Gastroenterology, Laboratory Diagnostic Medicine, and Orthopaedic Surgery, the First Affiliated Hospital of Chongqing Medical University, Chongqing 400016, China; Molecular Oncology Laboratory, Department of Orthopaedic Surgery and Rehabilitation Medicine, The University of Chicago Medical Center, Chicago, IL 60637, USA; Ministry of Education Key Laboratory of Diagnostic Medicine, and the School of Laboratory Medicine, Chongqing Medical University, Chongqing 400016, China; Departments of Nephrology, Gastroenterology, Laboratory Diagnostic Medicine, and Orthopaedic Surgery, the First Affiliated Hospital of Chongqing Medical University, Chongqing 400016, China; Molecular Oncology Laboratory, Department of Orthopaedic Surgery and Rehabilitation Medicine, The University of Chicago Medical Center, Chicago, IL 60637, USA; Molecular Oncology Laboratory, Department of Orthopaedic Surgery and Rehabilitation Medicine, The University of Chicago Medical Center, Chicago, IL 60637, USA; Department of Orthopaedic Surgery, Union Hospital of Tongji Medical College, Huazhong University of Science and Technology, Wuhan 430022, China; Division of Gastroenterology and Hepatology, Department of Medicine, University of Illinois at Chicago, Chicago, IL 60612, USA; Molecular Oncology Laboratory, Department of Orthopaedic Surgery and Rehabilitation Medicine, The University of Chicago Medical Center, Chicago, IL 60637, USA; Molecular Oncology Laboratory, Department of Orthopaedic Surgery and Rehabilitation Medicine, The University of Chicago Medical Center, Chicago, IL 60637, USA; Molecular Oncology Laboratory, Department of Orthopaedic Surgery and Rehabilitation Medicine, The University of Chicago Medical Center, Chicago, IL 60637, USA; Molecular Oncology Laboratory, Department of Orthopaedic Surgery and Rehabilitation Medicine, The University of Chicago Medical Center, Chicago, IL 60637, USA; Molecular Oncology Laboratory, Department of Orthopaedic Surgery and Rehabilitation Medicine, The University of Chicago Medical Center, Chicago, IL 60637, USA; Molecular Oncology Laboratory, Department of Orthopaedic Surgery and Rehabilitation Medicine, The University of Chicago Medical Center, Chicago, IL 60637, USA; Molecular Oncology Laboratory, Department of Orthopaedic Surgery and Rehabilitation Medicine, The University of Chicago Medical Center, Chicago, IL 60637, USA; Molecular Oncology Laboratory, Department of Orthopaedic Surgery and Rehabilitation Medicine, The University of Chicago Medical Center, Chicago, IL 60637, USA; Molecular Oncology Laboratory, Department of Orthopaedic Surgery and Rehabilitation Medicine, The University of Chicago Medical Center, Chicago, IL 60637, USA; Molecular Oncology Laboratory, Department of Orthopaedic Surgery and Rehabilitation Medicine, The University of Chicago Medical Center, Chicago, IL 60637, USA; Section of Plastic Surgery, Department of Surgery, The University of Chicago Medical Center, Chicago, IL 60637, USA; Molecular Oncology Laboratory, Department of Orthopaedic Surgery and Rehabilitation Medicine, The University of Chicago Medical Center, Chicago, IL 60637, USA; Section of Plastic Surgery, Department of Surgery, The University of Chicago Medical Center, Chicago, IL 60637, USA; Departments of Nephrology, Gastroenterology, Laboratory Diagnostic Medicine, and Orthopaedic Surgery, the First Affiliated Hospital of Chongqing Medical University, Chongqing 400016, China; Molecular Oncology Laboratory, Department of Orthopaedic Surgery and Rehabilitation Medicine, The University of Chicago Medical Center, Chicago, IL 60637, USA; Ministry of Education Key Laboratory of Diagnostic Medicine, and the School of Laboratory Medicine, Chongqing Medical University, Chongqing 400016, China; Molecular Oncology Laboratory, Department of Orthopaedic Surgery and Rehabilitation Medicine, The University of Chicago Medical Center, Chicago, IL 60637, USA; Section of Plastic Surgery, Department of Surgery, The University of Chicago Medical Center, Chicago, IL 60637, USA

## Abstract

MicroRNAs (miRNAs or miRs) are single-stranded, ∼22-nucleotide noncoding RNAs that regulate many cellular processes. While numerous miRNA quantification technologies are available, a recent analysis of 12 commercial platforms revealed high variations in reproducibility, sensitivity, accuracy, specificity and concordance within and/or between platforms. Here, we developed a universal hairpin primer (UHP) system that negates the use of miRNA-specific hairpin primers (MsHPs) for quantitative reverse transcription PCR (RT-qPCR)-based miRNA quantification. Specifically, we analyzed four UHPs that share the same hairpin structure but are anchored with two, three, four and six degenerate nucleotides at 3′-ends (namely UHP2, UHP3, UHP4 and UHP6), and found that the four UHPs yielded robust RT products and quantified miRNAs with high efficiency. UHP-based RT-qPCR miRNA quantification was not affected by long transcripts. By analyzing 14 miRNAs, we demonstrated that UHP4 closely mimicked MsHPs in miRNA quantification. Fine-tuning experiments identified an optimized UHP (OUHP) mix with a molar composition of UHP2:UHP4:UHP6 = 8:1:1, which closely recapitulated MsHPs in miRNA quantification. Using synthetic LET7 isomiRs, we demonstrated that the OUHP-based qPCR system exhibited high specificity and sensitivity. Collectively, our results demonstrate that the OUHP system can serve as a reliable and cost-effective surrogate of MsHPs for RT-qPCR-based miRNA quantification for basic research and precision medicine.

## INTRODUCTION

Mature microRNAs (miRNAs or miRs) are a group of evolutionarily conserved endogenous, single-stranded, small noncoding RNAs with an average length of 22 nucleotides (nt), ranging from 18 to 25 nt (1–4). The biogenesis of miRNAs starts with their transcription into primary miRNA (pri-miRNA) transcripts, which are subsequently processed into precursor miRNAs (pre-miRNAs) and finally into mature miRNAs through DROSHA/DICER cleavage machinery (3,4). Mechanistically, miRNAs are associated with Argonaute (AGO) proteins to form the so-called RNA-induced silencing complex and post-transcriptionally modulate gene expression by guiding AGOs to complementary regions of target mRNAs to repress their translation or regulate degradation (3,4). It has been shown that miRNAs exhibit tissue-specific expression patterns (3). Pri-miRNAs can generate a single mature miRNA or clusters of related miRNAs (3). Furthermore, miRNAs can be grouped into families based on the similarity of their seed sequences, which comprise nucleotides 2–8 (counting from the 5′ end) and are primarily responsible for miRNA targeting of mRNAs (3). Emerging evidence has shown that miRNAs are essential regulators of numerous key cellular processes, including apoptosis, proliferation or differentiation, and dysregulation of miRNAs may lead to the development of human diseases such as cancer and other chronic and metabolic disorders (3,4).

According to the world’s largest collection of miRNA data, the miRNA registry database miRBase (mirbase.org), the human genome encodes 2654 mature miRNAs (1908 in mice and 728 in rats) (miRBase v.22) (5), although GENCODE (v.29) documents >200 000 transcripts, including isoforms with slight variations (6). Another recently established miRNA candidate database miRCarta lists 12 857 human miRNA precursors (7). However, it has recently been reported that only ∼2300 true human mature miRNAs were extrapolated, 1115 of which are currently annotated in miRBase v.22 (8). The main reason that many miRNAs are not classified as ‘high confidence’ is the lack of expression data. Additionally, the abundance of different miRNAs in different cells and tissues varies drastically, from 0 to 1.4 × 10^5^ reads per million (5). In fact, 1225 human miRNAs (64%) do not have ≥20 reads associated with each arm in the datasets and thus cannot be confidently annotated (5).

Given the fact that miRNA expression levels vary significantly in different cells and tissues, accurate miRNA quantification is critical to assess biological functions and possible pathogenic roles of miRNAs. Ever since miRNAs were first discovered, numerous techniques have been devised to detect miRNA expression under various physiological and pathological conditions (9–12). In general, the miRNA detection methods can be divided into the following categories: (i) conventional techniques such as northern blotting (NB), microarray, *in situ* hybridization and quantitative reverse transcription PCR (RT-qPCR); (ii) biosensor techniques such as electrochemical-based detection, optical-based detection and nanotube-based techniques; and (iii) other emerging techniques, including next-generation sequencing (NGS), and nucleic acid amplification techniques such as rolling circle amplification (RCA), duplex-specific nuclease (DSN)-based amplification, loop-mediated isothermal amplification (LAMP), exponential amplification reaction (EXPAR) and strand-displacement amplification (SDA) (9–12).

Each of the above detection techniques has its unique advantages, as well as inherent shortcomings, including long processing time, laborious procedures, low throughput, large sample size requirements, false positivity, lack of sensitivity and/or costly instrument requirements. Not surprisingly, a comprehensive comparison analysis of the 12 commonly used commercial platforms for quantifying miRNA expression, including small RNA (sRNA) sequencing, RT-qPCR and microarray hybridization, revealed high variations in reproducibility, sensitivity, accuracy, specificity and concordance of differential expression within and/or between platforms (13). Nonetheless, RT-qPCR-based detection of miRNA expression remains one of the most commonly used methods ever since the introduction of stem–loop or hairpin primers for miRNA RT reactions (14), or the use of the poly(A) polymerase to polyadenylate mature miRNAs coupled with a poly(T) adapter to generate a cDNA (15). However, the hairpin or stem–loop primer system requires the use of miRNA-specific hairpin primers (MsHPs), which is not cost-effective and has low throughput.

In this study, we sought to develop a cost-effective and reliable universal hairpin primer (UHP) system that not only negates the use of MsHPs for RT reactions but also has high throughput potential. Specifically, we comprehensively analyzed a panel of four UHPs that share the same stem–loop/hairpin structure but anchored with two, three, four and six degenerate nucleotides at their 3′-ends (namely UHP2, UHP3, UHP4 and UHP6), and found that all four degenerate UHPs yielded robust RT products and specifically quantified individual miRNAs by qPCR with high efficiency similar to that of MsHPs. We also showed that the UHP-based RT-qPCR miRNA quantification was not affected by the presence of ribosomal RNAs and long transcripts. By analyzing a panel of 14 miRNAs, we demonstrated that, while still overestimating, the degenerate tetramer UHP4 closely mimicked MsHPs in RT-qPCR-based miRNA quantification. Interestingly, our results suggest that the hairpin-containing degenerate hexamer-initiated RT-qPCR analysis may overestimate the expression levels of coding and noncoding transcripts. Further fine-tuning experiments identified an optimized UHP (OUHP) mix with the molar composition of UHP2:UHP4:UHP6 = 8:1:1 that closely recapitulated MsHPs in miRNA quantification. It is conceivable that the OUHP system can be easily adapted for other forms of qPCR detection chemistry, and/or modified to implement multiplex miRNA quantification. Taken together, our results demonstrate that the reported OUHP system can serve as a best surrogate of any MsHP for RT-qPCR-based quantification of miRNA expression in a cost-effective and/or high-throughput fashion, which should be a valuable tool for basic research and precision medicine.

## MATERIALS AND METHODS

### Cell culture and chemicals

Human HEK-293, human osteosarcoma 143B and human melanoma A375 cells were obtained from the American Type Culture Collection (Manassas, VA). All cells were cultured in DMEM supplemented with 10% fetal bovine serum (Gemini Bio-Products), 100 U/ml penicillin and 100 μg/ml streptomycin at 37°C in 5% CO_2_ as described previously (16–19). Unless indicated otherwise, other chemicals were purchased from Thermo Fisher Scientific (Waltham, MA) or Millipore Sigma (St Louis, MO).

### Design and synthesis of MsHPs and UHPs for reverse transcription reactions

**Figure 1.**
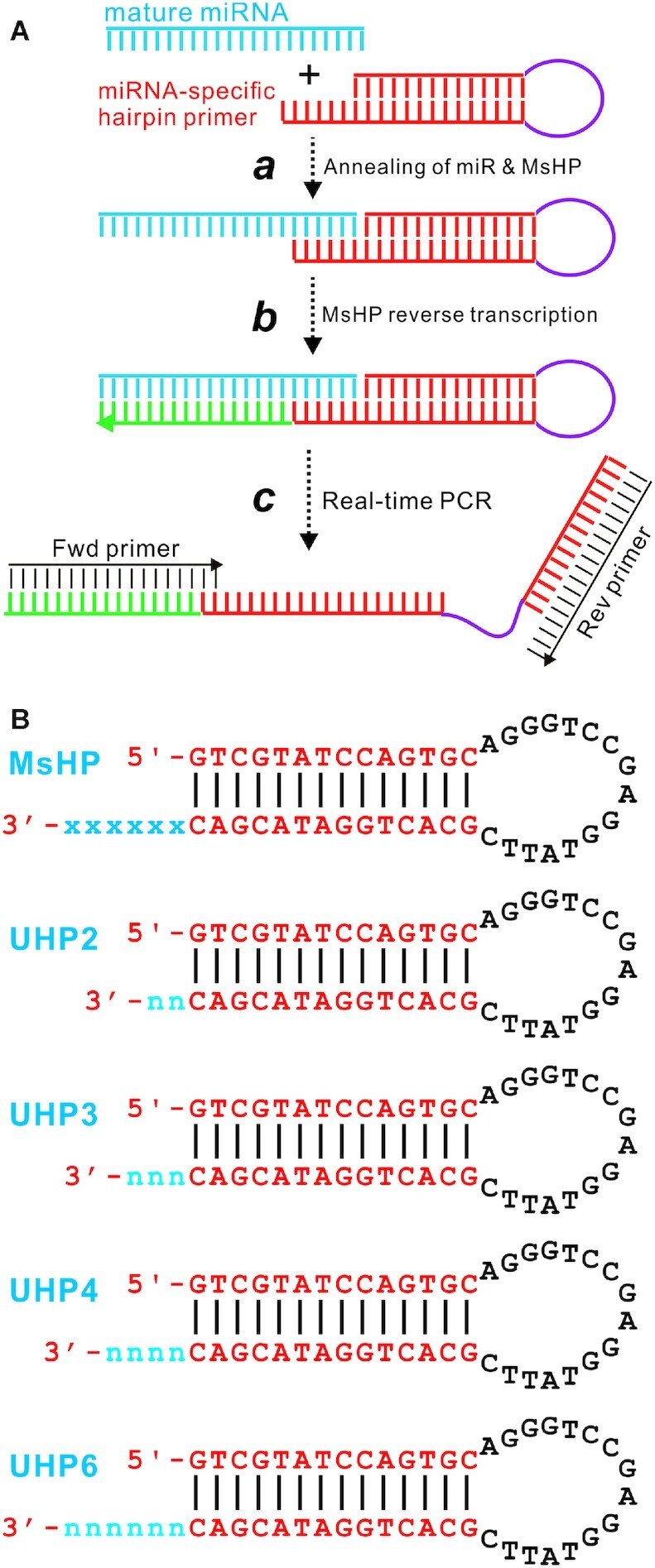
Schematic representation of the UHP system. (**A**) Schematics of conventional hairpin (or stem–loop) primer-based qPCR analysis of miRNA expression. An MsHP contains six nucleotides complementary to the 3′-end of mature miRNA, followed by a stem–loop structure. Once MsHP anneals to the targeted miRNA (**a**), RT reaction is carried out (**b**). The resultant RT product is used as a template for real-time quantitative PCR analysis (**c**) using a forward primer matching to the 5′-end of the mature miRNA and a reverse primer complementary to the 3′-end of the hairpin or stem–loop structure. (**B**) The schematic structure and nucleotide sequences of the tested UHPs. MsHP is a representative MsHP that contains a 14-bp stem, 16-nt loop and six nucleotides complementary to 3′-end of mature miRNA (indicated as ‘x’). UHP2, UHP3, UHP4 and UHP6 represent the four UHPs and share the same hairpin sequence as that of MsHPs, except that they contain two, three, four and six randomized nucleotides at the 3′-end of the stem sequence.

The design of hairpin or stem–loop primers for reverse transcription of miRNA samples is illustrated in Figure 1. All DNA oligonucleotides including qPCR primers were synthesized by Millipore Sigma. Synthetic mature miRNAs HSA-LET7d, HSA-LET7e, HSA-LET7i and HSA-LET7g were ordered from the Integrated DNA Technologies (Coralville, IA). The oligonucleotide sequences and their utilities are summarized in Supplementary Table S1.

### Total RNA isolation and sRNA (<200 nt) purification

Total RNA was isolated from exponentially growing HEK-293 cells using the NucleoZOL RNA Isolation Kit (Takara Bio USA, Mountain View, CA) according to the manufacturer’s instructions as described (20–22). To purify sRNA (<200 nt), we performed magnetic bead-based size selection with the commercially available Mag-Bind^®^ TotalPure NGS magnetic beads (Omega Bio-Tek, Inc., Norcross, GA) as described previously (23). Briefly, 5 μg of total RNA was dissolved in 20 μl RNase-free molecular biology grade ddH_2_O and mixed with 20 μl Mag-Bind beads (i.e. RNA/magnetic bead vol/vol ratio of 1:1). The RNA/magnetic bead mixture was incubated at room temperature for 10 min. The mixture was subjected to a magnet, and the sRNA (<200 nt)-containing supernatant was collected, while the large transcripts (>200 nt) bound to beads and were discarded. The collected sRNA was subjected to PC8 phenol/chloroform extraction, followed by ethanol precipitation. The recovered sRNA was dissolved in 20 μl RNase-free molecular biology grade ddH_2_O for reverse transcription reactions, or kept at −80°C.

### Characterization and quantification of the purified sRNA

After magnetic bead-based size selection, the recovered sRNA collection was assessed by using the Agilent 2100 Bioanalyzer (Santa Clara, CA) as described (24). Briefly, the recovered sRNA and total RNA samples (1.0 μl each) were loaded onto the Bioanalyzer RNA Nano Chips, along with size marker. The chip was subjected to electrophoresis according to the manufacturer’s instructions. The integrity and quantity of the RNA samples were visualized in both gel images and electropherograms.

### RT reactions using hairpin (stem–loop) primers

The 14 MsHPs and 4 UHPs were dissolved in RNase-free ddH_2_O at 1.0 μg/μl. The MsHP pool was created by mixing 10 μl of each MsHP. For RT reactions, 1 μg of total RNA or 0.1 μg of purified sRNA (in 10 μl ddH_2_O) was mixed with 2.0 μl of MsHP pool, or UHPs (i.e. UHP2, UHP3, UHP4 and UHP6), and annealed at 70°C for 5 min. After being cooled down on ice, each RNA/hairpin primer mixture was supplemented with 0.5 μl of RNase inhibitor (New England Biolabs, or NEB, Ipswich, MA), 2 μl of 10× RT buffer (NEB), 2 μl of 10 mM dNTPs, 0.5 μl of M-MuLV Reverse Transcriptase (NEB) and 3 μl RNase-free ddH_2_O. The RT reactions were kept at 25°C for 10 min, and then at 37°C for 30 min. Eighty microliters of ddH_2_O was added to the RT products, which served as qPCR templates with further dilutions and were kept at −80°C.

### Touchdown quantitative real-time PCR and data analysis

To increase the annealing temperature, a sequence of AGCC was added to the first 17 nt of all mature miRNAs, and used as miRNA qPCR forward primers. The oligonucleotide 5′-GTG CAG GGT CCG AGG TAT TC-3′, which is derived from the hairpin or stem–loop structure, was used as a common miRNA qPCR reverse primer. Primers for the reference transcript human 5S ribosomal RNA were designed using the Primer3Plus program. The touchdown quantitative real-time PCR (TqPCR) reactions were set up by using the 2× Forget-Me-Not™ EvaGreen qPCR Master Mix (Biotium, Fremont, CA), and carried out by using CFX-Connect (Bio-Rad) as previously described (25–28). The TqPCR cycling program was as follows: 95°C for 3 min for 1 cycle; 95°C for 20 min and 66°C for 10 min, for 4 cycles by decreasing 3°C per cycle; and 95°C for 20 min, 55°C for 10 min and 70°C for 1 min, followed by plate read, for 40 cycles.

Five-fold serial dilutions were performed to determine the amplification efficiency for each qPCR primer pair. No template control was used as a negative control. All reactions were done in triplicate. To quantitatively assess the quantification cycle (Cq) deviation from the MsHP group, ΔCq values were calculated for the UHP groups by subtracting individual average Cq value from respective Cq value for the MsHP group: ΔCq = average Cq (MsHP) − average Cq (UHP).

### Data analysis and statistical evaluation

All qPCR reactions were done in triplicate and/or in three independent batches of experiments. The linear mixed-effects models fitted by restricted maximum likelihood (REML) with the lme4 R package were employed to identify the fittest UHP, compared with the Cq values yielded by using MsHP. The nonparametric Kruskal–Wallis test with pairwise comparisons using the Wilcoxon rank sum exact test was carried out to assess the statistical difference among the ΔCq values of the four UHPs, relative to that of the MsHP group. Linear regression and correlation coefficient analysis were carried out to assess the effect of long transcripts on miRNA quantification. Whenever a comparison was made, a *P*-value <0.05 was considered statistically significant. All statistical analyses were performed using R statistical software (version 4.0.4, 2021; R Foundation for Statistical Computing, Vienna, Austria).

## RESULTS

### A novel UHP system provides a broad dynamic range of amplification in qPCR-based detection of miRNA expression

Since its development nearly two decades ago, the miRNA-specific stem–loop (or hairpin) primer-based RT-PCR method has been widely used to quantify miRNA expression (14). In this conventional system, the MsHP contains six nucleotides complementary to the 3′-end of mature miRNA, followed by a stem–loop structure (Figure 1A). Once MsHP anneals to the targeted miRNA, RT reaction is carried out and the resultant RT product is used as a template for real-time quantitative PCR analysis using a forward primer matching to the 5′-end of the mature miRNA and a reverse primer complementary to the 3′-end of the hairpin or stem–loop structure (Figure 1A).

While the MsHP system has been a robust system in miRNA quantification, it is not cost-effective for large-scale and/or high-throughput analysis of multiple miRNAs simultaneously. To overcome this limitation, we designed a novel UHP system for RT-PCR-based miRNA quantification (Figure 1B). In this system, we tested four UHPs, designated as UHP2, UHP3, UHP4 and UHP6, which share the same hairpin sequence as that of MsHPs, except that they contain two, three, four and six randomized nucleotides at the 3′-end of the stem sequence. Their hairpin structures are illustrated in Figure 1B.

**Figure 2.**
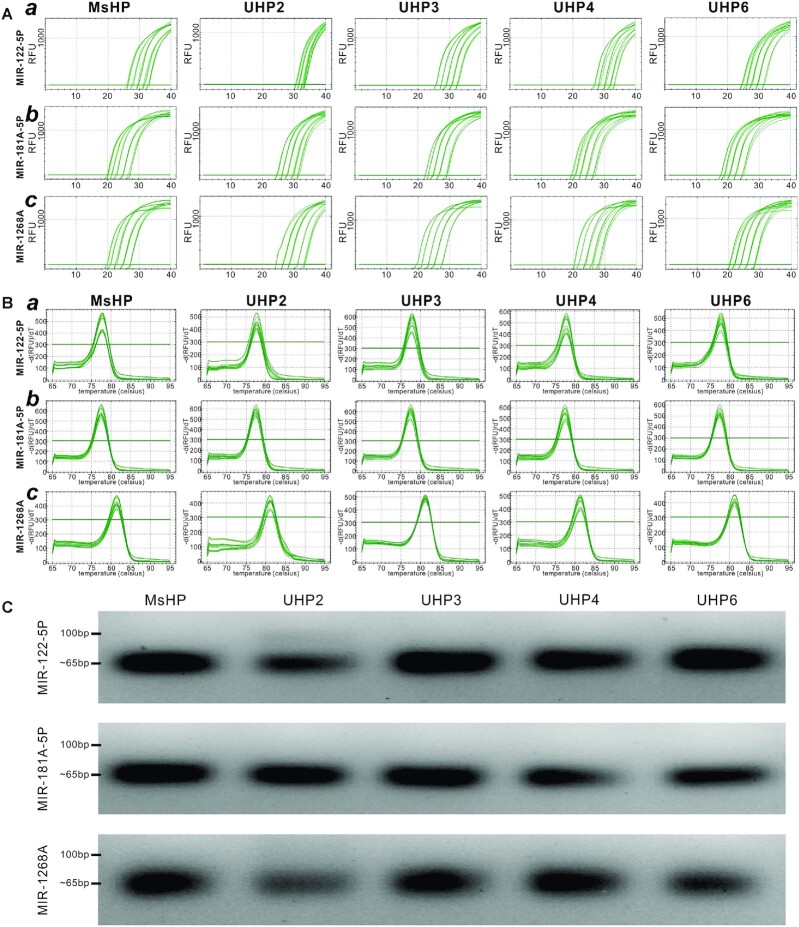
Sensitivity and specificity of the UHP-based qPCR analysis of miRNA expression in comparison with MsHPs. (**A**, **B**) Dynamic range and standard curve analysis of UHPs versus MsHP. UHP- and MsHP-derived RT products using total RNA from HEK-293 cells were subjected to 4-fold serial dilutions and used for TqPCR. Three representative miRNAs, HSAMIR-122-5P (**a**), HSAMIR-181A-5P (**b**) and HSAMIR-1268A (**c**), were selected for dynamic range of amplification (**A**) and melt curve analysis (**B**). Standard curves are shown in Supplementary Figure S1. (**C**) Amplification specificity. The qPCR end products with expected sizes of ∼65 bp were assessed by electrophoresis on 2% agarose gels. Only the results from the 1:160 dilution groups (the second dilution for the three miRNAs) are shown.

We first tested the sensitivity and specificity of the four UHPs as RT primers, in comparison with those of the MsHP pool. The RT products were prepared with the four UHPs and MsHP, and then 4-fold serially diluted. For practical reasons, we selected three representative miRNAs, HSA-MIR-122-5P (Figure 1A, panel a), HSA-MIR-181A-5P (Figure 1A, panel b) and HSA-MIR-11268A (Figure 1A, panel c), and quantified their expression in the prepared RT products. We found that the three selected miRNAs displayed proper amplification curves in a template concentration-dependent fashion (Figure 2A, panels a–c). However, it is noteworthy that, when compared with the MsHP group, the amplification curves for the UHP2 group were right-shifted, while the amplification curves for the UHP6 group were left-shifted, at least for HSA-MIR-122-5P and HSA-MIR-181A-5P (Figure 1A, panels a and b). Nonetheless, the UHPs yielded excellent standard curves for the three miRNAs tested with *R*^2^ value >0.97, except for MIR-122-5P primed with UHP2 (*R*^2^ value = 0.711) (Supplementary Figure S1A, panels a–c). The melt curves indicate that all UHPs generated a single peak (Figure 2B), and agarose gel analysis also confirmed that all UHP groups generated a single band with the same size as that of the MsHP groups (Figure 2C). Alternatively, we performed a serial dilution of total RNA, followed by reverse transcription using MsHP and the four UHP primers. The RT products were subjected to TqPCR analysis using specific forward primers for HSA-MIR-122-5P, HSA-MIR-181A-5P and HSA-MIR-1268A. Our results demonstrated that the amplification curves for the UHP2 group were right-shifted, while the amplification curves for the UHP6 group were left-shifted, at least for HSA-MIR-122-5P and HSA-MIR-181A-5P (Supplementary Figure S1B, panels a–c). Collectively, these results demonstrate that (i) the four UHPs were effective and specific in initiating the RT reactions for miRNA quantification and (ii) the miRNA qPCR primer pairs consisting of miRNA-specific forward primers and the common reverse primer derived from the hairpin provided a reasonable dynamic range of detection with high amplification efficiency.

### The degenerate tetramer UHP4 closely recapitulates MsHP pool in miRNA qualification

**Figure 3.**
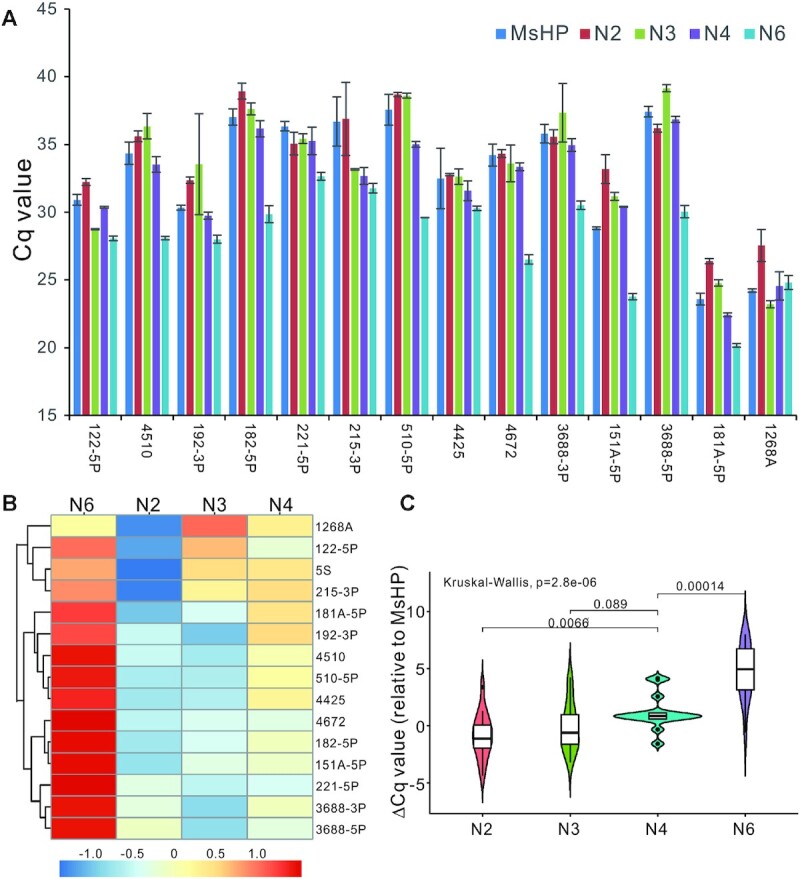
Validation of the tetramer UHP4 as the ‘winning’ universal primer among the tested four UHPs. (**A**) Cq value comparison of the four UHPs relative to MsHP. RT products prepared with the MsHP and the four UHPs using total RNA from HEK-293 cells were subjected to qPCR analysis of the indicated 14 miRNA expression. The average Cq values were calculated and plotted. N2 = UHP2, N3 = UHP3, N4 = UHP4 and N6 = UHP6. (**B**) The heatmap and cluster analysis of ΔCq value relative to MsHP for each UHP. The ΔCq value was calculated by subtracting each UHP’s average Cq value from respective MsHP’s Cq value. 5S RNA was included as an internal reference transcript. (**C**) The box and whisker plot of ΔCq value relative to MsHP for each UHP. The nonparametric Kruskal–Wallis test was carried out to assess the statistical difference among the four UHPs.

As shown earlier, while the four UHPs were able to detect miRNA expression with high sensitivity and specificity, it is important to determine whether their amplifications represent the actual expression levels of the tested miRNAs as defined by their MsHPs. To ensure the validity of such fit test assays, we chose a panel of 14 miRNAs with a wide range of expression levels. The RT products were prepared from total RNA samples with the MsHP pool, UHP2, UHP3, UHP4 and UHP6 primers, and subjected to TqPCR as previously described (25), using the 14 miRNA-specific forward primers and a common reverse primer. For the RT products derived from MsHPs and four UHPs, 5 of the 14 analyzed miRNAs exhibited the Cq values relatively close to those of the respective MsHPs, including HSAMIR-122-5p, HSAMIR-192-3p, HSAMIR-221-5p, HSAMIR-4425 and HSAMIR-1268A (Figure 3A). However, the Cq values of the remaining nine miRNAs had significant deviations from those of the respective MsHPs, and in particular, the UHP6 group seemingly yielded significantly lower Cq values, compared with respective MsHPs (Figure 3A). Furthermore, we conducted the linear mixed-effects models fitted by REML and identified that UHP4 yielded Cq values that were the closest to those of respective MsHPs.

We further calculated the ΔCq values relative to respective MsHPs for the UHPs. Heatmap clustering analysis indicated that 13 of the 14 tested miRNAs have positive ΔCq values in the UHP6 group, indicating an overestimation of miRNA expression compared to that of respective MsHPs (Figure 3B). Conversely, 11 of the 14 tested miRNAs have negative ΔCq values in the UHP2 group, suggesting that the miRNA expression may be underestimated in this group, compared with that of the MsHPs (Figure 3B). For the UHP3 group, while 9 of the 14 miRNAs have negative ΔCq values and 5 have positive ΔCq values, the range of the ΔCq values is significantly narrower, and 10 of the 14 miRNAs have the ΔCq values within ±2 range (Figure 3B). Consistent with the conclusion of the linear mixed-effects model fit test, the UHP4 group yields the smallest overall ΔCq values, and 11 of the 14 miRNAs have the ΔCq values of <1.0, compared with those of respective MsHPs (Figure 3B), suggesting that UHP4 may be the best surrogate for MsHPs in RT-PCR-based miRNA quantification.

We also analyzed the ΔCq data using the box and whisker plot. The nonparametric Kruskal–Wallis analysis indicates that there was a statistical difference among the four UHPs (*P*-value = 2.8e−6). As shown in Figure 3C, the medians (shown in the middle quartile) for UHP2, UHP3 and UHP4 were close to ‘0’, while the median for UHP6 deviated significantly from ‘0’ (Figure 3C). As expected, the UHP4 group yielded the tightest box of the middle 50%, and the median was the closest to ‘0’ among the four UHP groups, whereas the whiskers were the shortest among the four UHP groups (Figure 3C), indicating lower variabilities outside the upper and lower quartiles than other UHP groups. Interestingly, the difference in data distributions between UHP3 and UHP4 was not statistically significant (Figure 3C). Collectively, these results demonstrate that UHP4 is the most approximate of the tested MsHPs in RT-PCR-based miRNA quantification.

### The presence of ribosomal RNAs and long transcripts does not affect the UHP-based qPCR quantification of miRNA expression

**Figure 4.**
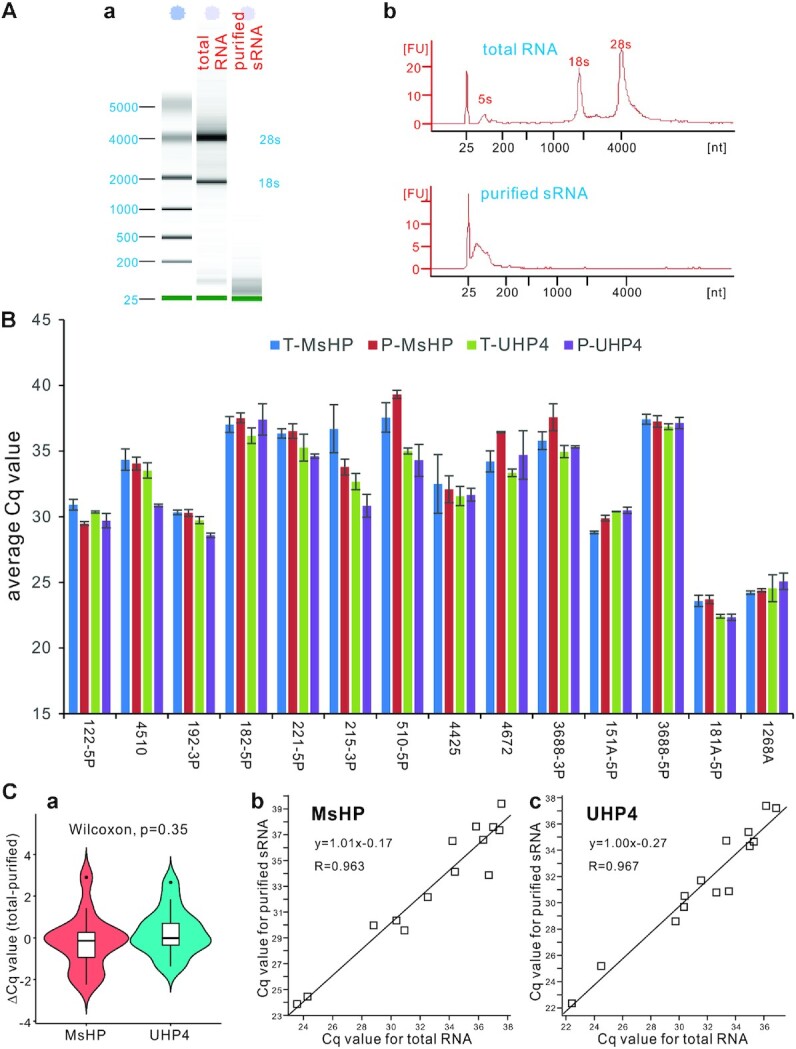
The effect of large transcripts on miRNA quantification on the UHP-based qPCR system. (**A**) Removal of large transcripts from total RNA using size selection magnetic beads. Total RNA from HEK-293 cells was mixed with Mag-Bind beads at vol/vol ratio of 1:1 to isolate sRNAs (i.e. <200 nt). The purified sRNA was assessed by an Agilent 2100 Bioanalyzer, and the results were visualized in both gel images (**a**) and electropherograms (**b**). (**B**) Average Cq values of the 14 tested miRNAs in total RNA versus purified sRNA samples for RT reactions using MsHP or UHP4. T-MsHP and T-UHP4 indicate the RT products of the total RNA sample prepared with MsHP and UHP4 primers, respectively. P-MsHP and P-UHP4 indicate the RT products of the purified sRNA sample prepared with MsHP and UHP4 primers, respectively. (**C**) The box and whisker plot, linear regression and correlation coefficient analysis of miRNA detection in total RNA versus purified sRNA. Linear regression and correlation of the average Cq value correlations between total RNA and purified sRNA samples using MsHP (**b**) or UHP4 (**c**) were also analyzed.

Many miRNA quantification protocols require the purification of sRNAs using commercially available kits. In this study, we used the 3′-end randomized hairpin primers or UHPs for RT reactions. It is conceivable that the UHPs may produce large amounts of non-miRNA-related RT products from rRNAs and long transcripts and lead to decreased sensitivity and specificity in miRNA quantification. To test whether such adverse effect may exist, we conducted a side-by-side comparison study of miRNA quantification by using the RT products prepared from total RNA and purified sRNA samples. We employed our recently validated protocol to separate different sizes of nucleic acids through the commercially available size selection magnetic bead system (23,24), and removed RNA species >200 nt (Figure 4A, panel a). The recovered sRNAs were <200 nt based on the results from Agilent 2100 Bioanalyzer assays (Figure 4A, panel b).

Using the purified sRNA sample along with its corresponding total RNA sample, we performed RT reactions using MsHP and UHP4 primers. The average Cq values of the 14 miRNAs were at similar levels, while certain variations were observed in a few miRNAs, albeit without statistical significance (*P*> 0.20) (Figure 4B). The box and whisker plot analysis indicated that the ΔCq values between total RNA samples and purified sRNAs for the 14 tested miRNAs were tightly centered at the ‘0’ position, and the nonparametric Wilcoxon signed rank test found no statistical difference (*P* = 0.35) (Figure 4C, panel a). Furthermore, linear regression and correlation coefficient analysis indicated that average Cq values of the 14 tested miRNAs were highly correlated between total RNA and purified sRNA samples for both MsHP and UHP4 primer groups (Figure 4C, panels b and c). We further examined the effect of large RNA transcripts on the UHP-based qPCR quantification of miRNA expression in the RNA samples isolated from another three cell lines, HEK293, A375 and 143B cells. When the magnetic bead-based size selection RNA (<200 nt) and total RNA were subjected to RT reactions using MsHP, we found that the expression of the five tested miRNAs did not show any statistical difference between the purified sRNA groups and total RNA groups (Supplementary Figure S2A–C). Taken together, these results demonstrate that the presence of ribosomal RNAs and long transcripts does not significantly affect the UHP-based RT-qPCR quantification of miRNA expression in biological samples.

### Identification and characterization of an OUHP cocktail that serves as a faithful surrogate of MsHPs in high-throughput miRNA quantification

**Figure 5.**
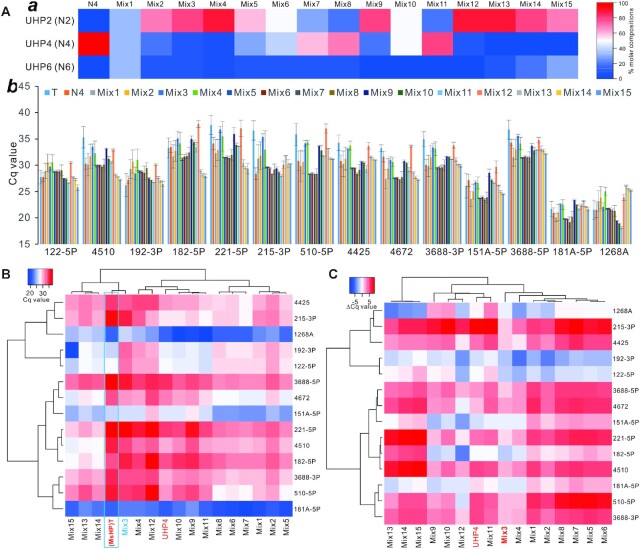
Characterization and identification of the OUHP cocktail mixtures as potential MsHP surrogates. (**A**) Compositions of the 15 UHP mixtures of UHP2, UHP4 and UHP6 at various molar percentages, and the UHP4 as a reference control (**a**). The Cq values of the analyzed 14 miRNAs with the 15 UHP mixtures, along with MsHP (T) and UHP4 (N4) RT products using total RNA from HEK-293 cells (**b**). (**B**) Heatmap analysis of the Cq values of the analyzed 14 miRNAs with the 15 UHP mixtures, along with MsHP (T) and UHP4 groups. The heatmap was generated by using the complete linkage clustering method with Spearman rank correlation as the distance measurement method. The MsHP (T) group is boxed, while Mix3 and UHP4 groups are highlighted. (**C**) Heatmap analysis of the ΔCq values of the analyzed 14 miRNAs with the 15 UHP mixtures, along with the UHP4 group. The heatmap was generated by using the complete linkage clustering method with Spearman rank correlation as the distance measurement method. The UHP4 group is highlighted. The ΔCq value was calculated as follows: ΔCq = Cq (MsHP) – Cq (UHP mix).

While the results presented in Figure 3 indicate that the tetramer UHP4 closely recapitulated the Cq values obtained from the MsHP-initiated RT products, UHP4 still tended to overestimate miRNA expression in general. In order to develop an OUHP to serve as a faithful MsHP surrogate, we formed a panel of 15 UHP formulations, namely Mix1 through Mix15, by mixing UHP2, UHP4 and/or UHP6 at various molar compositions in percentages (Figure 5A, panel a; Supplementary Figure S3A). We subsequently assessed the resultant Cq values for 14 miRNAs in comparison with those of respective MsHPs (Figure 5A, panel b). Heatmap clustering analysis of the Cq values of the four tested miRNAs revealed that Mix3 was clustered together with MsHP, while Mix4 and Mix12 were clustered closely with UHP4 (Figure 5B).

We further analyzed the ΔCq values relative to MsHPs for the 14 tested miRNAs by the 14 cocktail mixtures, as well as by UHP4. Heatmap clustering analysis of the ΔCq values indicated that the Mix3 group yielded the smallest deviations from zero among all 15 cocktail groups and the UHP4 group, while most of the other groups tended to significantly overestimate the levels of miRNA expression (Figure 5C). A direct plot of the ΔCq values also revealed that the Mix3 group displayed the smallest fluctuations around the ‘zero’ axis (Supplementary Figure S3B), which was further confirmed by boxplot analysis (Supplementary Figure S4). Interestingly, the distributions and variations of the ΔCq values between Mix3 and UHP4 were statistically significant, suggesting that UHP4 may be less optimal than Mix3 in representing MsHPs in miRNA quantification. Collectively, these results strongly suggest that Mix3 (i.e. UHP2:UHP4:UHP6 = 8:1:1, also designated as the OUHP) may serve as the best surrogate of MsHP for quantifying miRNA expression in a high-throughput fashion.

Lastly, we analyzed the detection efficiency and specificity of the OUHP system using the exemplary isomiR LET7 miRNA family (Supplementary Figure S5A, panels a and b). We first demonstrated that the OUHP primers effectively detected the expression of all eight members of the LET family with similar efficiency, compared with those of respective LET7-specific hairpin primers and the pooled LET7 family-specific hairpin primers (Supplementary Figure S5B). Using the synthetic mature LET7d and LET7i, we showed that the OUHP primers detected a broad dynamic range of the mature LET7d and LET7i (Supplementary Figure S5C, panels a and b). Furthermore, when the synthetic mature LET7e, LET7g and LET7i were subjected to OUHP RT reaction, followed by qPCR analysis with LET7-specific forward primers, we found that the Cq values were significantly lower in the synthetic LET7-specific forward primer group than those of other LET7 forward primer groups (Supplementary Figure S5D, panels a–c). Furthermore, the relative expression calculated for possible ‘cross-reactivity’ from other LET7 family member forward primers was <0.1% of the respective ‘perfect match’ counterpart in most cases (Supplementary Figure S5D, panel d). Collectively, these results demonstrate that the OUHP primer system can provide significant detection specificity for even closely related miRNA family members.

## DISCUSSION

The increasing recognition of miRNAs’ biological functions in regulating many aspects of cellular processes mandates readily available technologies to quantify miRNA expression. These detection systems should be reliable, sensitive, easy to use and cost-effective. For the past two decades, numerous techniques have been developed to assess miRNA expression levels (9–12). The conventional NB technique was first used for the initial discovery of miRNA lin-4 in 1993 (1), and remains the only technique that allows for the quantitative visualization of miR (9). The NB technique was later modified by labeling DNA probes with 3′-digoxigenin hapten to avoid the use of radioisotopes, and/or by using locked nucleic acid in nucleic acid probes to improve sensitivity and match specificity (9). However, compared with other detection methods, NB suffers from low sensitivity, time-consuming, low throughput and large RNA quantity requirement. Similar to NB, miRNA microarray analysis relies on the sensitive, specific hybridization of the target miR to its complementary DNA probe, which is spatially organized on a solid phase or gene chip, and visualized with fluorescence or imaging instrumentation. Microarray analysis of miRNA expression represents one of the earliest techniques capable of high-throughput and massive parallel analysis of numerous miRNAs in one sample at the same time. The drawbacks of the microarray method include relatively higher cost, limited dynamic range of detection, semiquantitative nature of detection, secondary validation requirement and limited specificity on closely related miRNA sequences.

In recent years, NGS has become a viable technique to quantify miRNA expression (9–12,29). Other emerging detection techniques include various biosensor techniques involved in electrochemical-based detection, optical-based detection and nanotube-based methodology, and nucleic acid amplification techniques such as RCA, DSN-based amplification, LAMP, EXPAR and SDA (9–12). Each of these detection techniques has its unique advantages, as well as inherent shortcomings, including long processing time, laborious procedures, low throughput, large sample size requirements, false positivity, lack of sensitivity and/or costly instrument requirements.

Given the advantages in detection sensitivity, high throughput potential and technical ease, RT-qPCR analysis is the most popular method to detect and quantify miRNA expression (9–12,30). The first use of a qPCR-based method for miRNA quantification was described in 2004, in which two forward primers and one reverse primer were used to detect the expression of pri- and pre-miRNA levels (31). However, the qPCR-based detection approach has to overcome at least two technical challenges: the short length of mature miRNAs (∼22 nt) and high similarity of multiple members of miRNA families.

Numerous efforts have been devoted to increasing miRNA length at the RT stage, primarily focusing on two approaches: poly(A) tailing and the use of stem–loop/hairpin adaptors/primers (9–12,30). The former approach involves the use of poly(A) polymerase-mediated polyadenylation, a poly(T) adapter and an miRNA-specific forward primer (15). A variation of the poly(A) tailing approach was to use T4 RNA ligase to uniformly extend miRNAs’ 3′-ends by adding a linker adapter, which then served as an ‘anchor’ to prime cDNA synthesis and throughout qPCR to amplify specifically target amplicons (32). The use of stem–loop or hairpin primers for miRNA RT reactions followed by TaqMan PCR analysis was also introduced in 2005 (14), although several modifications, including the use of universal TaqMan probe and longer stem–loop RT primers, were reported (33,34). A recently reported stem–loop variation called dumbbell PCR method took advantage of the T4 RNA ligase 2-mediated ligation of either 5′- or 3′-end stem–loop adapter to target miRNAs (35). While most of these RT-qPCR-based methods provide high sensitivity and specificity for miRNA quantification, these systems require the use of MsHPs, which is not cost-effective, time-consuming and/or has low throughput.

In this study, we sought to develop a UHP system that would overcome the necessity of using miRNA-specific primers for RT reactions, not only being cost-effective but also rendering the system with high throughput potential. By analyzing a panel of four hairpin primers with two to six degenerate nucleotides at their 3′-ends, we demonstrated that the degenerate tetramer hairpin primer (i.e. UHP4) yielded RT-qPCR quantification results closely mimicking those of MsHPs for the 14 tested miRNAs although the UHP4-based RT-qPCR analysis still overestimated miRNA expression. Meanwhile, we found that, based on the Cq values for the 14 tested miRNAs, the degenerate dimer hairpin primer (i.e. UHP2) tended to underestimate miRNA expression, whereas the degenerate hexamer hairpin primer (i.e. UHP6) overestimated miRNA expression. These features of the degenerate UHPs indicated that it was possible to develop an optimal mixture of the degenerate dimer, tetramer and hexamer UHPs as a reliable surrogate of MsHPs for miRNA quantification. By analyzing a panel of 15 cocktail mixtures of UHP2, UHP4 and/or UHP6, we identified the optimal UHP mix (also known as OUHP) of UHP2:UHP4:UHP6 = 8:1:1 mole ratio, which best recapitulated the MsHP pool in miRNA quantification. Interestingly, a degenerate octamer ‘universal stem–loop primer’ at the 3′-end of the conventional stem–loop primer was used for reverse transcription and to ensue miRNA quantification (36). However, the octamer RT primer was mostly validated on a synthetic miR-155, and the sensitivity of the octamer stem–loop primer system was shown to be significantly lower than that of MsHPs (36). Thus, unlike our extensively validated OUHP system, it is unclear whether the degenerate octamer stem–loop primer may be useful for general miRNA quantification.

It is also noteworthy that our study demonstrated that the degenerate hexamer hairpin primer-initiated RT-qPCR analysis may overestimate the true expression levels of coding and noncoding transcripts. While in this study we only carried out SBYR Green-based qPCR analysis, the OUHP system should be readily adapted for other forms of qPCR detection chemistry, such as TaqMan, cycling probe technology, molecular beacons and minor groove binding probes. Moreover, it is conceivable that with certain modifications of the hairpin sequence and detection chemistry, the OUHP system can be upgraded to implement multiplex analysis of miRNA expression. Nonetheless, while the OUHP RT-qPCR system has great promise in streamlining miRNA quantification with high throughput potential, this system has some inherent limitations. First, the base-pairing feature of the degenerate nucleotides at the 3′-end of OUHP prevents it from discriminating isomiRs, miRNAs with similar seed sequences. Second, the reported validation studies mostly focused on mature miRNAs, so it is yet to be validated whether the OUHP system can be used to determine pre-miRNA and/or pri-miRNA expression levels.

In summary, we comprehensively analyzed a panel of four UHPs with two, three, four and six degenerate nucleotides at their 3′-ends (namely UHP2, UHP3, UHP4 and UHP6), and demonstrated that the degenerate tetramer hairpin primer (i.e. UHP4), while still overestimating, closely mimicked MsHPs in RT-qPCR-based quantification of miRNA expression. Interestingly, our results suggest that the hairpin-containing degenerate hexamer-initiated RT-qPCR analysis may overestimate the expression levels of coding and noncoding transcripts. Further fine adjustments identified an OUHP with the molar composition formulation of UHP2:UHP4:UHP6 = 8:1:1. Collectively, our results demonstrate that the OUHP system can serve as a best surrogate of MsHPs for RT-qPCR-based quantification of miRNA expression in a cost-effective and/or high-throughput fashion, which should be a valuable resource for basic research and precision medicine.

## Supplementary Material

gkab1153_Supplemental_File
