# Supplementary material for: OUHP: an optimized universal hairpin primer system for cost-effective and high-throughput RT-qPCR-based quantification of microRNA (miRNA) expression
Source: Nucleic Acids Res. 2021 Nov 25;50(4):e22. doi: 10.1093/nar/gkab1153 (PMC8887422; doi:10.1093/nar/gkab1153)
Supplement: gkab1153_Supplemental_File [file gkab1153_supplemental_file.pdf]

Table S1. List of Oligonucleotides Used in the Study

Table S1. List of Oligonucleotides Used in the Study

HAS-MIR	Sequence	Use
hsa-miR-122-5p	AGCCTGGAGTGTGACAATGGT	qPCR Forward
hsa-miR-4510	AGCCTGAGGGAGTAGGATGTA	
hsa-miR-192-3p	AGCCCTGCCAATTCCATAGGT	
hsa-miR-182-5p	AGCCTTTGGCAATGGTAGAAC	
hsa-miR-221-5p	AGCCACCTGGCATACAATGTA	
hsa-miR-215-3p	AGCCTCTGTCATTTCTTTAGG	
hsa-miR-510-5p	AGCCTACTCAGGAGAGTGGCA	
hsa-miR-4425	AGCCTGTTGGGATTCAGCAGG	
hsa-miR-4672	AGCCTTACACAGCTGGACAGA	
hsa-miR-3688-3p	AGCCTATGGAAAGACTTTGCC	
hsa-miR-151a-5p	AGCCTCGAGGAGCTCACAGTC	
hsa-miR-3688-5p	AGCCAGTGGCAAAGTCTTTCC	
hsa-miR-181a-5p	AGCCAACATTCAACGCTGTCG	
hsa-miR-1268a	AGCCCGGGCGTGGTGGTGGGG	
hsa-let-7d-5p	AGAGGTAGTAGGTTGCATA	
hsa-let-7e-5p	TGAGGTAGGAGGTTGTATA	
hsa-let-7f-1-5p	TGAGGTAGTAGATTGTATA	
hsa-let-7a-1-5p	TGAGGTAGTAGGTTGTATA	
hsa-let-7c-5p	TGAGGTAGTAGGTTGTATG	
hsa-let-7b-5p	TGAGGTAGTAGGTTGTGTG	
hsa-let-7g-5p	TGAGGTAGTAGTTTGTACA	
hsa-let-7i-5p	TGAGGTAGTAGTTTGTGCT	
hsa-miR-122-5p	GTCGTATCCAGTGCAGGGTCCGAGGTATTCGCACTGGATACGACCAAACA	RT
hsa-miR-4510	GTCGTATCCAGTGCAGGGTCCGAGGTATTCGCACTGGATACGACAACCAT	
hsa-miR-192-3p	GTCGTATCCAGTGCAGGGTCCGAGGTATTCGCACTGGATACGACCTGTGA	
hsa-miR-182-5p	GTCGTATCCAGTGCAGGGTCCGAGGTATTCGCACTGGATACGACAGTGTG	
hsa-miR-221-5p	GTCGTATCCAGTGCAGGGTCCGAGGTATTCGCACTGGATACGACAAATCT	
hsa-miR-215-3p	GTCGTATCCAGTGCAGGGTCCGAGGTATTCGCACTGGATACGACTATTGG	
hsa-miR-510-5p	GTCGTATCCAGTGCAGGGTCCGAGGTATTCGCACTGGATACGACGTGATT	
hsa-miR-4425	GTCGTATCCAGTGCAGGGTCCGAGGTATTCGCACTGGATACGACATGGTC	
hsa-miR-4672	GTCGTATCCAGTGCAGGGTCCGAGGTATTCGCACTGGATACGACTGCCTC	
hsa-miR-3688-3p	GTCGTATCCAGTGCAGGGTCCGAGGTATTCGCACTGGATACGACAGAGTG	
hsa-miR-151a-5p	GTCGTATCCAGTGCAGGGTCCGAGGTATTCGCACTGGATACGACACTAGA	
hsa-miR-3688-5p	GTCGTATCCAGTGCAGGGTCCGAGGTATTCGCACTGGATACGACATATGG	
hsa-miR-181a-5p	GTCGTATCCAGTGCAGGGTCCGAGGTATTCGCACTGGATACGACACTCAC	
hsa-miR-1268a	GTCGTATCCAGTGCAGGGTCCGAGGTATTCGCACTGGATACGACCCCCCA	
hsa-let-7a/7d7/7e/7f	GTCGTATCCAGTGCAGGGTCCGAGGTATTCGCACTGGATACGACAACTAT	
hsa-let-7b	GTCGTATCCAGTGCAGGGTCCGAGGTATTCGCACTGGATACGACAACCAC	
hsa-let-7c	GTCGTATCCAGTGCAGGGTCCGAGGTATTCGCACTGGATACGACAACCAT	
hsa-let-7g	GTCGTATCCAGTGCAGGGTCCGAGGTATTCGCACTGGATACGACAACTGT	
hsa-let-7i	GTCGTATCCAGTGCAGGGTCCGAGGTATTCGCACTGGATACGACAACAGC	

Figure S1. Standard curve analysis of UHPs

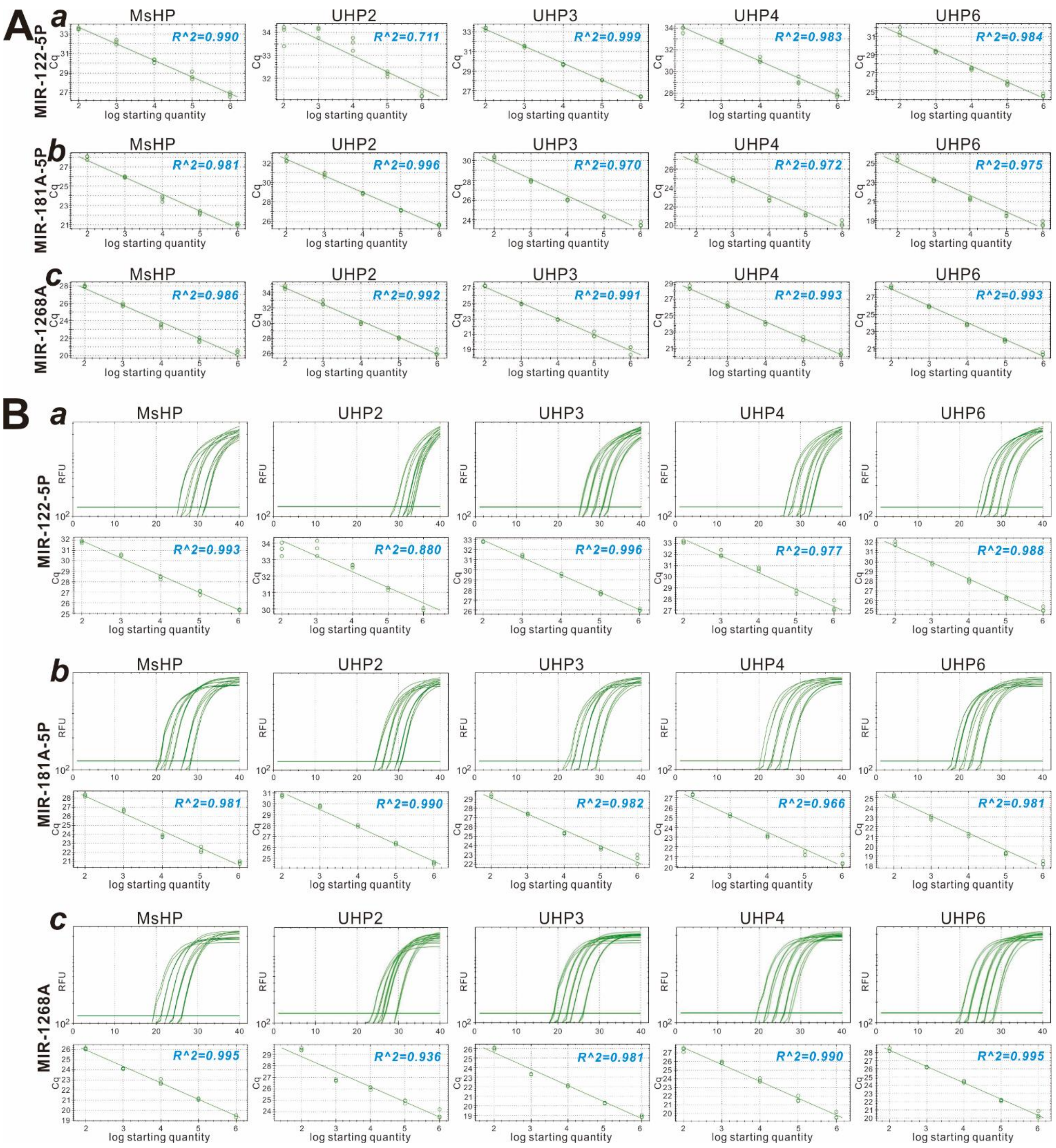

Figure S1. Standard curve analysis of UHPs. (**A**) RT products prepared by using MsHP and four UHP primers were 4-fold serially diluted and subjected to TqPCR analysis using specific forward primers for HSA-MIR-122-5P (***a***), HSA-MIR-181A-5P (***b***), and HSA-MIR-1268A (***c***). The standard curves were generated from the obtained Cq values. Linear regression analysis was performed and correlation coefficients were determined. (**B**) Total RNA (1µg/µl) from HEK-293 cells was 4-fold serially diluted and subjected to reverse transcription using MsHP and four UHP primers, followed by TqPCR analysis using specific forward primers for HSA-MIR-122-5P (***a***), HSA-MIR-181A-5P (***b***), and HSA-MIR-1268A (***c***). Dynamic range of amplification and standard curve analysis were carried out as described in (**A**).

Figure S2. The effect of large transcripts on miRNA qPCRs

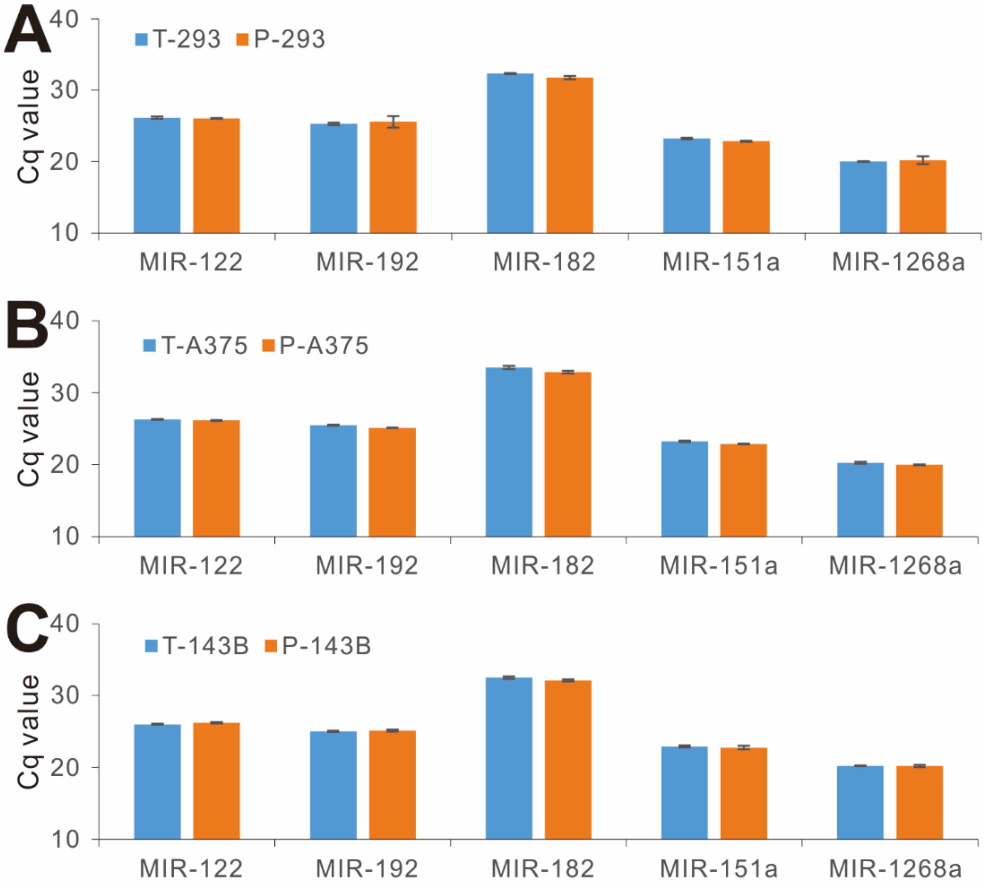

Figure S2. The effect of large transcripts on miRNA qPCRs. Total RNA was isolated from HEK293 (**A**), A375 (**B**) and 143B (**C**) cells, and subjected to magnetic bead-based size selection to remove RNA species >200nt. The resulting RNA samples were designated as P-293, P-A375 and P-143B, as opposed to their control counterparts T-293, T-A375 and T-143B. These RNA samples were subjected to RT reactions using MsHP, followed by qPCR analysis of the five miRNAs. All qPCR reactions were done in triplicate.

Figure S3. List of the tested 15 UHP cocktail mixtures and Distribution of the ΔCq values for the 15 tested UHP mixtures

A

Compositions (% in molar compositions) of the UHP Mixtures

	**N4**	Mix1	Mix2	**Mix3**	Mix4	Mix5	Mix6	Mix7	Mix8	Mix9	Mix10	Mix11	Mix12	Mix13	Mix14	Mix15
UHP2	**0**	33.33	70	**80**	90	60	50	30	20	80	50	20	90	90	80	70
UHP4	**100**	33.33	20	**10**	5	30	40	60	70	20	50	80	10	0	0	0
UHP6	**0**	33.33	10	**10**	5	10	10	10	10	0	0	0	0	10	20	30

B

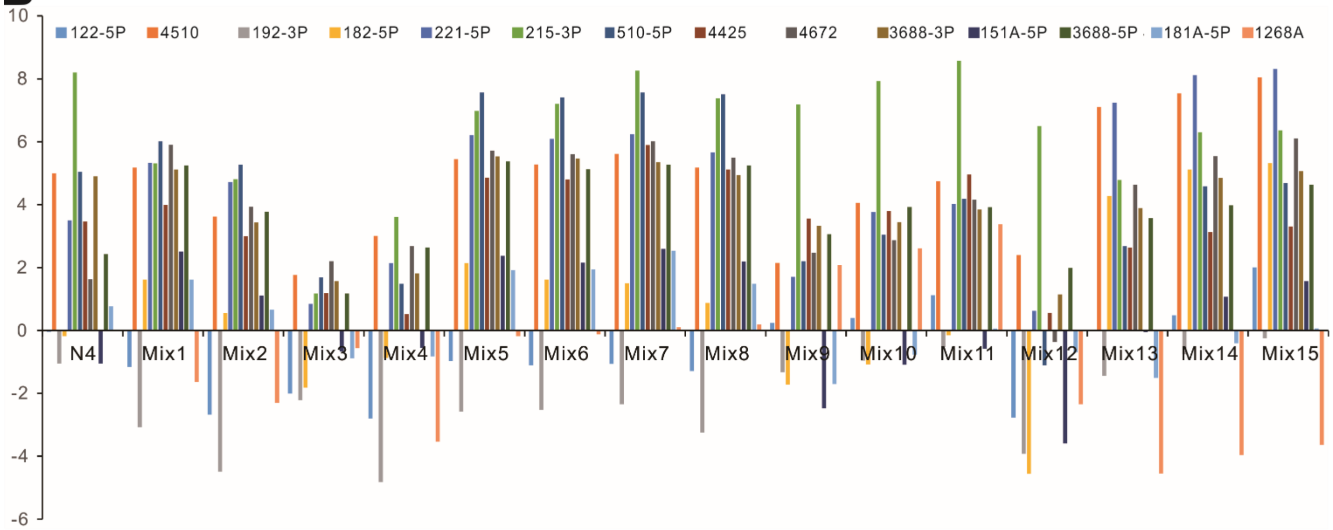

Figure S3. (A) List of the tested 15 UHP cocktail mixtures in molar compositions (%). **(B)** Distribution of the ΔCq values for the 15 tested UHP mixtures. Positive ΔCq values indicate lower Cq values in the UHP groups than that in the MsHP group, suggesting overestimation; vice versa for the negative ΔCq values.

Figure S4. Box plot analysis of ΔCq values of the 15 tested UHP mixtures

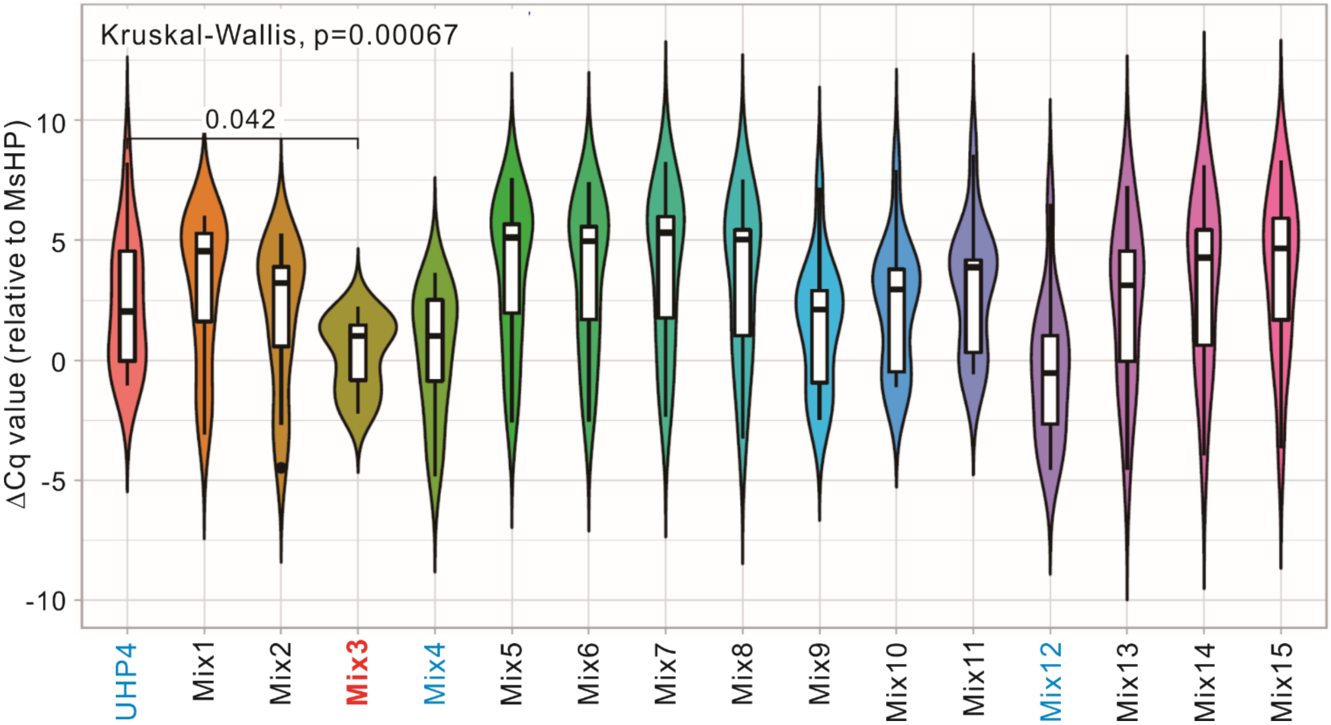

Figure S4. Box plot analysis of ΔCq values of the 15 tested UHP mixtures. Positive ΔCq values indicate lower Cq values in the UHP groups than that in the MsHP group, vice versa for the negative ΔCq values.

Figure S5. Detection specificity analysis of the LET7 family

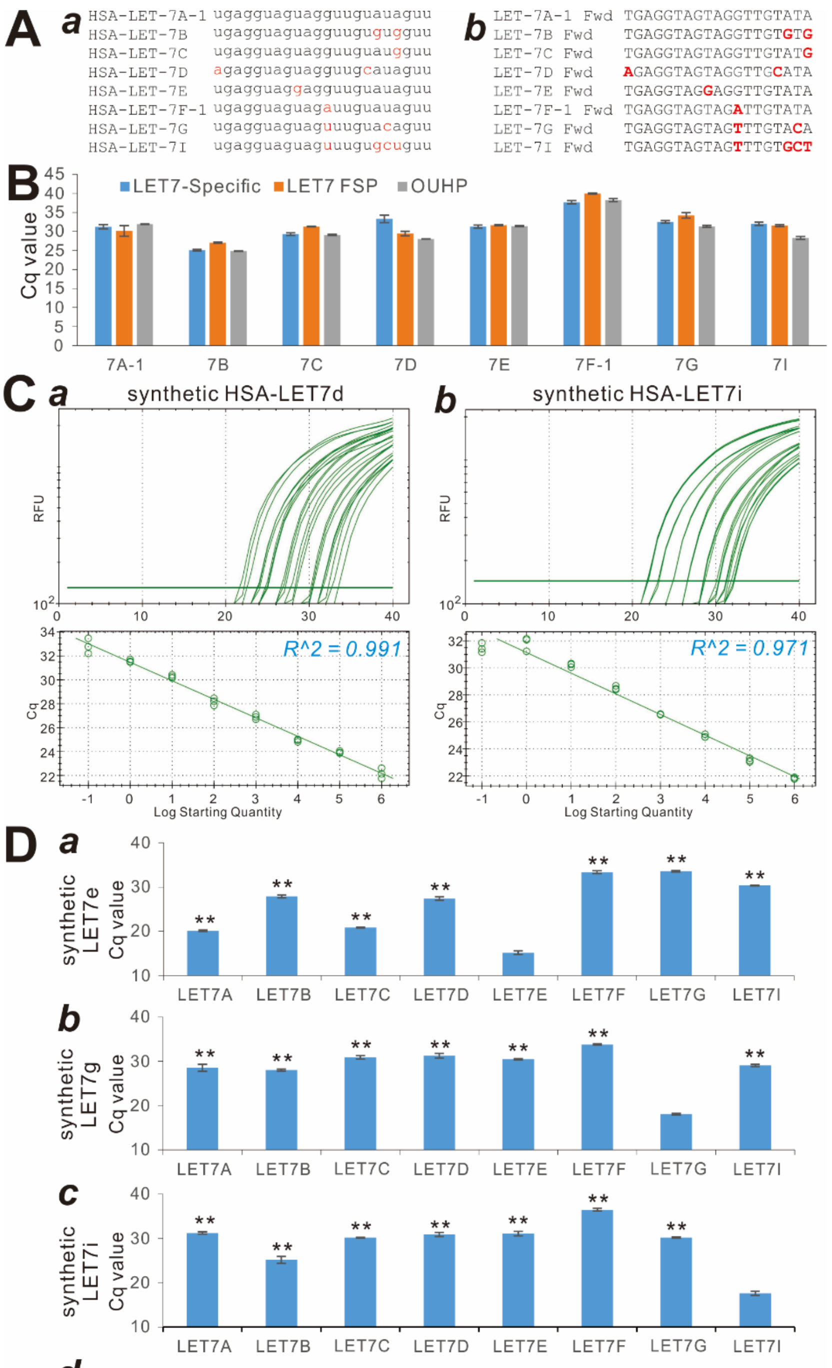

d

	Relative miR expression (% of MSP)							
	LET7A	LET7B	LET7C	LET7D	LET7E	LET7F	LET7G	LET7I
LET7E MSP	3.18900	0.01472	1.95854	0.02079	100	0.00033	0.00029	0.00268
LET7G MSP	0.06973	0.09861	0.01353	0.01060	0.01796	0.00179	100	0.04804
LET7I MSP	0.00802	0.53827	0.01659	0.01002	0.00867	0.00021	0.01632	100

Figure S5. Detection specificity analysis of the LET7 family. (A) Sequences for the mature miRNAs of the LET7 family members (a) and the LET7 member-specific forward primers. **(B)** Total RNA was subjected to RT reactions using the individual LET7-specific HP (LET7-specific), the pooled LET7 family-specific HP (LET7 FSP), or OUHP, followed by qPCR analysis with LET7-specific forward primers. **(C)** Dynamic amplification range and standard curve analyses. Ten nanograms (ng) of the synthetic mature miRNAs LET7d (***a***) and miR LET7i (***b***) were subjected to 4-fold serial dilutions, followed by RT reactions using OUHP and qPCR analysis with forward primers for LET7d (***a***) and LET7i (***b***), respectively. **(D)** Specificity of detection analysis. The synthetic mature miRNAs LET7e (***a***), LET7g (***b***), and LET7i (***c***) (about 100ng) were subjected to RT reaction with OUHP and qPCR analysis with LET-specific forward primers. "**" $p < 0.01$, compared with the average Cq value obtained with the forward primer for the respective synthetic LET7 mature miRNA. Relative expression (% relative to MSP) of individual LET7 was calculated using $2^{-\Delta\Delta Cq}$, whereas the respective "perfect match" group was set to 100% (***d***).
